# Mathematical Analysis of the Effect of Rotor Geometry on Cup Anemometer Response

**DOI:** 10.1155/2014/537813

**Published:** 2014-07-03

**Authors:** Ángel Sanz-Andrés, Santiago Pindado, Félix Sorribes-Palmer

**Affiliations:** ^1^Instituto Universitario de Microgravedad “Ignacio Da Riva” (IDR/UPM), Universidad Politécnica de Madrid, ETS de Ingeniería Aeronáutica y del Espacio, Plaza del Cardenal Cisneros 3, 28040 Madrid, Spain; ^2^Departamento de Infraestructura, Sistemas Aeroespaciales y Aeropuertos, Universidad Politécnica de Madrid, ETS de Ingeniería Aeronáutica y del Espacio, Plaza del Cardenal Cisneros 3, 28040 Madrid, Spain

## Abstract

The calibration coefficients of two commercial anemometers equipped with different rotors were studied. The rotor cups had the same conical shape, while the size and distance to the rotation axis varied. The analysis was based on the 2-cup positions analytical model, derived using perturbation methods to include second-order effects such as pressure distribution along the rotating cups and friction. The comparison with the experimental data indicates a nonuniform distribution of aerodynamic forces on the rotating cups, with higher forces closer to the rotating axis. The 2-cup analytical model is proven to be accurate enough to study the effect of complex forces on cup anemometer performance.

## 1. Introduction

### 1.1. Wind Speed Anemometry as an Important Tool in Wind Energy Generation

The importance of accuracy in wind speed measurements is emphasized as the wind energy sector is highly concerned with both wind turbine performance control and wind energy forecast on the field [1, 2]. The aforementioned accuracy of the measurements directly affects wind energy production, as this production is proportional to the third power of the wind speed [3].

On the other hand, it can be said that today the wind energy sector represents the larger demand of anemometers in the market, despite the increasing use of anemometers in other industries/applications. In addition, it seems that the demand of accurate anemometers will remain strong, although the investment in the wind energy industry has decreased in the traditionally leaders of the sector (Germany, Spain, and Denmark), new players are now very active (China, USA, India, and Brazil) [4].

Finally, the cup anemometer is at present the standardized instrument included in the most relevant code of practice regarding wind turbine power performance measurements (IEC 61400-12-1) [5].

### 1.2. Cup Anemometer Aerodynamics

A cup anemometer can be studied from two different perspectives: as a meteorological instrument or as a body in autorotation. As a meteorological instrument, the cup anemometer has been studied for a long time, using different techniques and mathematical models, under different climatic conditions and focusing on particular aspects of their performance and response. In addition to these, it should also be said that some important research projects regarding cup anemometers have been carried out based on public funds [6–9].  Table 1 summarizes some of these aspects of cup anemometers, along with the authors of the corresponding research contributions (an extensive review of the available literature has been included in the table).

In many cases, the purpose of the research conducted throughout the twentieth century involved studying certain characteristics of anemometer performance to obtain experimental data in order to develop mathematical models. It must be underlined that a validated mathematical model to predict anemometer performance under normal working conditions is a very useful and important tool in different fields, such as meteorology and the wind energy industry.

**Figure 1:**
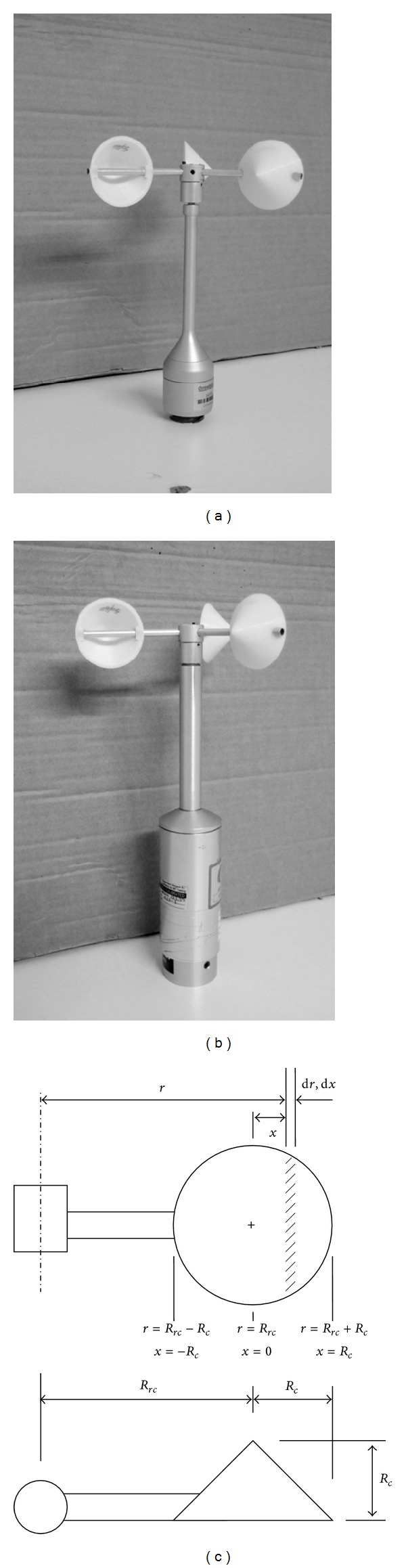
Anemometers used in the testing campaign related to the calibration results from Table 2, Climatronics 100075 (b) and Ornytion 107A (a). A sketch of the conical cups of the rotors tested is also included (c).

Mathematical models normally include Euler's equation for describing the rotation of a rigid body (anemometer cup rotor), affected by both aerodynamic and friction torque [5]:

(1) $$\begin{array}{c} I\frac{\text{d}\omega }{\text{d}t}=M_{A}+M_{f}, \end{array}$$

where *ω* is the rotational speed of the anemometer rotor, *I* is the moment of inertia, *M*_*A*_ is the aerodynamic torque, and *M*_*f*_ is the frictional torque that depends on the air temperature, *T*, and the rotation speed, *ω* (from [10]: *M*_*f*_ = *B*_0_(*T*) + *B*_1_(*T*)*ω* + *B*_2_(*T*)*ω* ^2^, where coefficients *B*_0_, *B*_1_, and *B*_2_ are negative (the friction torque, *M*_*f*_, in expression (1) has a negative sign in the updated version of [5]. Therefore, coefficients *B*_0_, *B*_1_, and *B*_2_ of the friction torque expression will be positive, if this is taken into account)). The frictional torque, *M*_*f*_, is generally neglected in all mathematical models [10], as it is very low compared to the aerodynamic torque within the normal wind speed range [11, 12]. The aerodynamic torque is modeled as a function that includes the wind speed, *V*, the cup center rotation speed, *ωR*_*rc*_ (*R*_*rc*_ is the cup center rotation radius, see Figure 1), and the vertical component of the wind speed, *w*. This function is derived using nondimensional and perturbation analysis to the second-order Taylor polynomial, where the coefficients are measured by means of carefully planned testing [11, 13–19]. This method was therefore developed to correlate a group of parameters with a specific individual anemometer, in order to obtain the highest possible accuracy in the predictions relating to dynamic behavior (i.e., accelerations and decelerations of the rotor from a steady situation, at constant rotational speed). This procedure requires no aerodynamic model of the rotating body or rotor.

However, a properly developed aerodynamic model of the rotor can be very interesting, as the effect of certain parameters can be identified to provide a better understanding of cup anemometer performance. Some efforts have been made to integrate rotational aerodynamics in the mathematical models with interesting results [14, 20–24]. Nevertheless, it is fair to say that, due to the simplifications in the development of these models, some deviations remain when compared to the experimental results [21, 22].

The aim of this paper is to continue with the postprocessing of the experimental results from the systematic cup anemometer testing campaign carried out at the IDR/UPM Institute during 2011 and to derive a simple mathematical model for studying some aspects of cup anemometer performance. The testing campaign consisted of 21 calibrations performed on 2 different anemometers (Climatronics 100075 and Ornytion 107A, see Figure 1), while varying the cup radius, *R*_*c*_, and the cup center rotation radius, *R*_*rc*_, of the rotor (more information regarding the testing campaign can be found in [21]).  Table 2 includes the calibration results from that campaign (details concerning the calibration facility are included in Appendix A). These results are the slope and offset of the transfer function (commonly known as calibration constants *A* and *B* [25, 26]):

(2) $$\begin{array}{c} V=A\cdot f+B. \end{array}$$

This transfer function, obtained experimentally in a wind tunnel, relates the wind speed to the output frequency of the anemometer, *f* (see Appendix A). The slope of the transfer function given in terms of rotational frequency, *f*_*r*_, instead of output frequency, *f*:

(3) $$\begin{array}{c} V=A_{r}\cdot f_{r}+B, \end{array}$$

is also included in Table 2. This new slope, *A*_*r*_, is the result of multiplying calibration constant *A* by the number of pulses per turn, *N*_*p*_, given by the anemometer. (The Climatronics 100075 and Ornytion 107A anemometers give 30 and 2 pulses per turn, respectively [20].) Finally, the coefficients d*A*_*r*_/d*R*_*rc*_, d*B*/d*R*_*rc*_, *A*_*r*0_, and *B*_0_, (slope and offset) of the linear fittings with regard to the calibration coefficients, *A*_*r*_ and *B*, as a function of the cup center rotation radius, *R*_*rc*_

(4) Ar=dArdRrcRrc+Ar0,

(5) B=dBdRrcRrc+B0,

calculated for rotors with cups of the same radius, *R*_*c*_, are included in Table 2, along with the aforementioned results. The above equations were important for deriving the following experimentally fitted expressions [21]:

(6) Ar=dArdRrcRrc−Sc(ζ+ηSc−ξ),

(7) B=(ε+ϕSc−γ)Rrc−μSc−ψ.

The most relevant conclusions reached were as follows.

- The slope of the calibration transfer function, *A*_*r*_, depends on two different contributions, one related to the cup center rotation radius, *R*_*rc*_, and the other related to the cups' front area, *S*_*c*_, or cup radius, *R*_*c*_  (*S*_*c*_ = *πR*_*c*_^2^). The slope of expression (6), d*A*_*r*_/d*R*_*rc*_, seems to be directly related to the aerodynamic nondimensional coefficient of the cups, as very small differences in this coefficient were observed among the 42 calibrations performed on the 2 anemometers tested. That is, the fitting coefficient d*A*_*r*_/d*R*_*rc*_ did not seem to depend on the anemometer, with the same value for both the Climatronics 100075 and the Ornytion 107A anemometers, whereas the other fitting coefficients, *ζ*, *η*, and *ξ*, were different depending on the anemometer tested.
- The offset of the calibration transfer function, *B*, also depends on the same shape parameters, although in this case each contribution is not totally independent from the next. In this case, all the fitting parameters, *ε*, *γ*, *ϕ*, *μ*, and *ψ*, were different depending on the anemometer tested.

In the research described, cup anemometer performance was analyzed using the anemometer factor, *K*, which relates the wind speed, *V*, to the cup center rotation speed, *ωR*_*rc*_; that is, *K* = *V*/*ωR*_*rc*_. In the calculations of this factor from the calibration results, the offset constant, *B*, is usually neglected because it is small compared to the wind speed within the normal working range of the anemometer (average values of calibration constant *B* for three Class-1 anemometers are 0.179 m s^−1^ (Risø P2546A), 0.248 m s^−1^ (Thies Clima 4.3350), and 0.184 m s^−1^ (Vector Instruments A100 L2) [20]. The calibration range of an anemometer, according to MEASNET is from 4 m s^−1^ to 16 m s^−1^ [25], although sometimes the upper limit of this calibration range is larger [20]). Nevertheless, the anemometer factor does indeed depend on the offset constant:

(8) $$\begin{array}{c} K=\frac{V}{\omega R_{rc}}=\frac{A_{r}f_{r}+B}{2\pi f_{r}R_{rc}}=\frac{A_{r}}{2\pi R_{rc}}\frac{1}{1-\left(B/V\right)}. \end{array}$$

Moreover, in a recent study at the IDR/UPM Institute, differences of up to 13.4% were observed at a wind speed of *V* = 4 m/s regarding the anemometer factor, *K*, if the offset constant is taken into account [4]. To avoid this possible source of misunderstandings, a simplified anemometer factor, *K*_*S*_, is proposed in this paper:

(9) $$\begin{array}{c} K_{S}=\frac{V-B}{\omega R_{rc}}=\frac{A_{r}}{2\pi R_{rc}}. \end{array}$$

**Figure 2:**
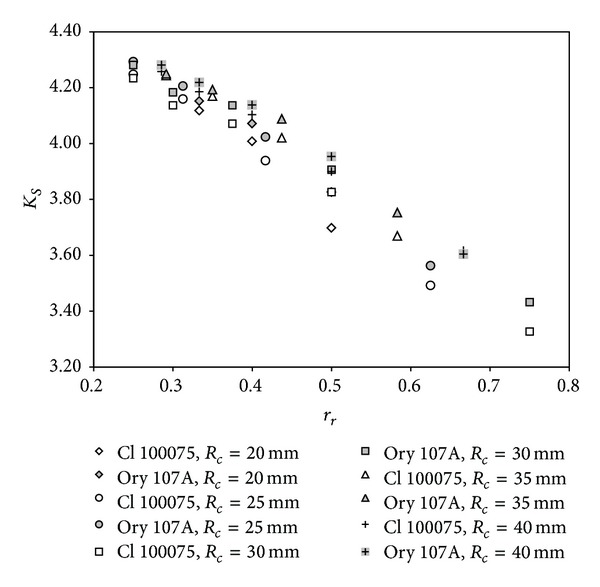
Simplified anemometer factor, *K*_*S*_, as a function of the ratio of the cup radius to the cup center rotation radius, *r*_*r*_ (*r*_*r*_ = *R*_*c*_/*R*_*rc*_), regarding the calibrations performed on the Climatronics 100075 anemometer (Cl 100075; white symbols) and the Ornytion 107A anemometer (Ory 107A; grey symbols) [21]. Both were equipped with *R*_*c*_ = 20 mm cups (rhombi), *R*_*c*_ = 25 mm cups (circles), *R*_*c*_ = 30 mm cups (squares), *R*_*c*_ = 35 mm cups (triangles), and *R*_*c*_ = 40 mm cups (crosses).

Figure 2 includes the simplified anemometer factor (hereinafter, anemometer factor), *K*_*S*_, calculated with the results from Table 2. It must also be said that in previous studies [4, 22], the anemometer factor was regarded as displaying quadratic behavior as a function of the ratio of cup radius to cup center rotation radius, *r*_*r*_ = *R*_*c*_/*R*_*rc*_. This consideration was based on the assumption of asymptotic behavior for low values of the aforementioned parameter (*r*_*r*_ → 0). This seems reasonable because in this case the analytical models showed a lower dependence on *r*_*r*_ [22]. However, the linear fittings to the data from Figure 2 show rather high determination coefficients, *R* ^2^, with minimum and average values of *R* ^2^ = 0.965 and *R* ^2^ = 0.985, respectively. This indicates linear rather than quadratic behavior within the normal range of parameter *r*_*r*_. Also, taking into account expressions (6) and (9), the following equation can be derived:

(10) $$\begin{array}{c} K_{S}=\frac{1}{2\pi }\left[\frac{\text{d}A_{r}}{\text{d}R_{rc}}-\left(\zeta^{\prime }R_{c}+\frac{\eta^{\prime }}{R_{c}^{2\xi -1}}\right)r_{r}\right], \end{array}$$

which is in agreement with the linear behavior mentioned above. Obviously, in the expression above, *ζ*′ and *η*′ are derived from the fitting coefficients *ζ* and *η* in (6).

To develop a mathematical model, the aerodynamic torque should be considered proportional to the dynamic pressure relative to the cup. On the other hand, because a dynamic problem is considered, the aerodynamic torque should be described in terms of a nondimensional parameter, Ω = *ωR*_*rc*_/*V*, formed by the rotational speed, *ω*, which is inversely proportional to the characteristic time of the movement, and the residence time, *VR*_*rc*_, comprised the wind speed, *V*, and a characteristic length of the rotor, *R*_*rc*_. This parameter, Ω, is also called the cup center nondimensional velocity.

**Figure 3:**
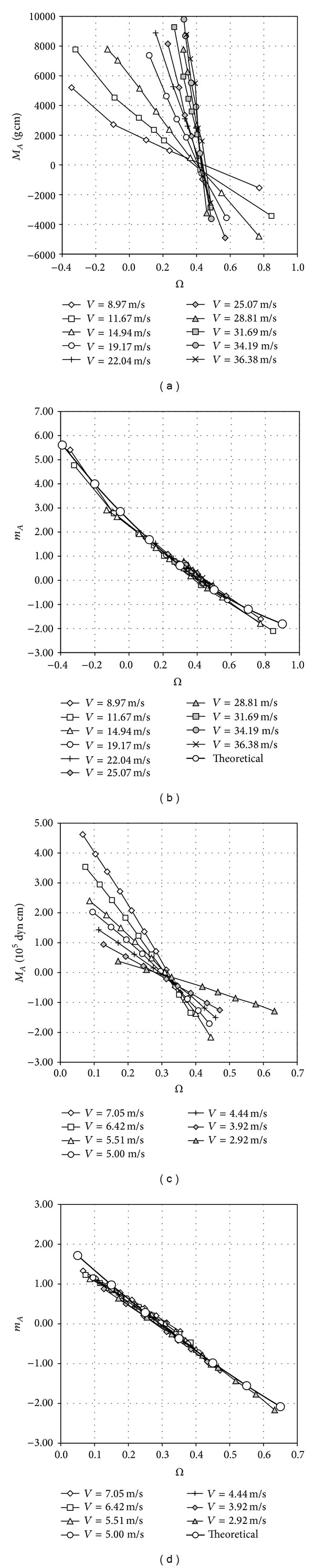
Aerodynamic torque, *M*_*A*_, measured on 4-cup [27] (a) and 3-cup [13] (c) anemometers as a function of the ratio between the cup center velocity and the wind speed, Ω (Ω = *ωR*_*rc*_/*V*). The nondimensional torque, *m*_*A*_, calculated for each curve, has also been included ((b) and (d)), together with the results from the classical theoretical model fitted to the curves; see expression (12).

In this regard, Figure 3 shows the experimental results from Brevoort and Joyner [27] and Wyngaard et al. [13]. In these graphs, the aerodynamic torque, *M*_*A*_, is shown as a function of the dimensionless parameter Ω. The nondimensional aerodynamic torque, *m*_*A*_ (see expression (12)), is also shown. It can be observed that the nondimensional curves tend to collapse into a single curve, revealing the relationship between the aforementioned dimensionless parameters, *m*_*A*_ and Ω. This behavior was also analyzed by Pedersen [10], who found a second-order polynomial relationship between the nondimensional aerodynamic torque, *m*_*A*_, and the speed ratio (the speed ratio is defined in [5] as *V* and *V*_*t*_, which are the wind speed at the calibration facility and the threshold velocity, respectively. The threshold velocity is derived by subtracting the friction effects from the calibration offset, *B*),  *λ* = *ωR*_*rc*_/(*V* − *V*_*t*_).

**Figure 4:**
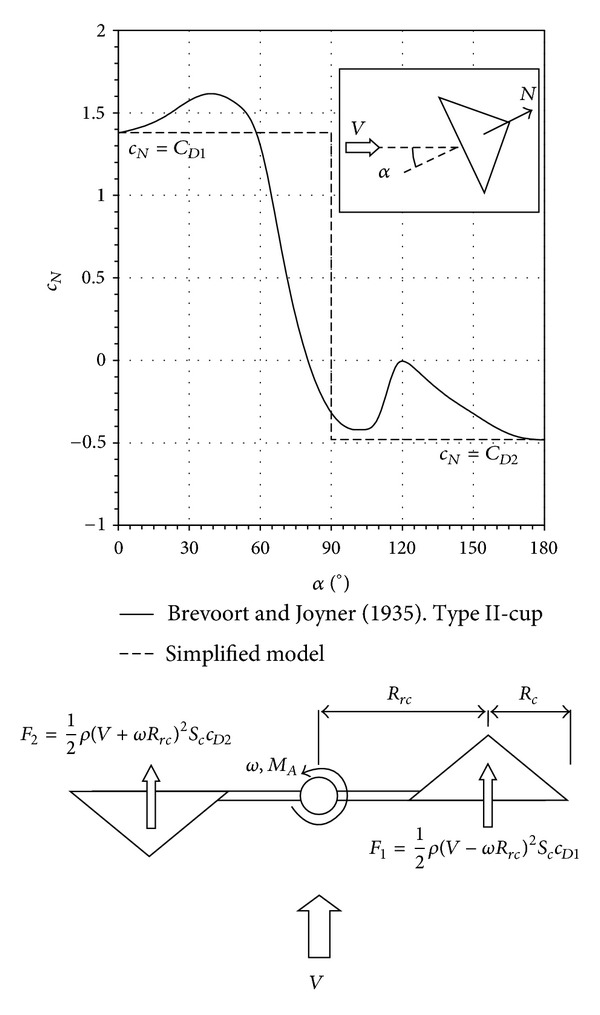
Normal aerodynamic force coefficient, *c*_*N*_, of the Brevoort and Joyner Type-II cup [27], plotted as a function of the wind direction with respect to the cup, *α*. The approximation used in the classical simplified model for aerodynamic torque on the anemometer rotor (expression (11)) is also included as a dashed line. See in the attached sketch the idealized approximation to the equilibrium point of the 2-cup analytical model, where *M*_*A*_ = 0,  *F*_1_ = *F*_2_, and, therefore, the rotation speed *ω* is constant.

The classical model for aerodynamic torque is represented by the following expression:

(11) MA=12ρScRrcNc ×[(V−ωRrc)2c1CD1−(V+ωRrc)2c2CD2],

where *ρ* is the air density, *N*_*c*_ the number of cups, *S*_*c*_ the front area of the cups, *V* the wind speed, *ω* the rotational speed, *C*_*D*1_ (concave side) and *C*_*D*2_ (convex side) are the aerodynamic drag force coefficients of the cups, and *c*_1_ and *c*_2_ are coefficients that take into account the effectiveness of the aerodynamic simplification. The average torque produced by each cup is then expressed as a function of the forces at only two positions. See Figure 4 for the normal-to-the-cup aerodynamic coefficient, *c*_*N*_, with regard to a nonrotating cup expressed as a function of the wind direction, *α*. The dashed line in the figure represents the approximation of aerodynamic torque produced by one cup shown in expression (11). That expression can be rewritten in nondimensional form:

(12) mA=MA(1/2)ρScRrcV2=Nc[(1−Ω)2c1CD1−(1+Ω)2c2CD2]=Ncc1CD1[(1−Ω)2−kD2(1+Ω)2],

where the dependence on the nondimensional parameter Ω is reflected. Also, a new parameter is included in the equation, the drag coefficient ratio, *k*_*D*_,

(13) $$\begin{array}{c} k_{D}={\left(\frac{c_{2}C_{D2}}{c_{1}C_{D1}}\right)}^{1/2}. \end{array}$$

With this model, the equilibrium point, *m*_*A*_ = 0, is obtained for

(14) $$\begin{array}{c} \Omega =\Omega_{0}=\frac{1-k_{D}}{1+k_{D}}. \end{array}$$

Theoretical equation (12) has been fitted to the graphs for the nondimensional torque included in Figure 3. The results are in good correspondence with the experimental results, indicating the validity of this analytical approach. The simplification made with (11) is also known as the 2-cup analytical model, which models the anemometer's behavior with a rotor consisting of *N*_*c*_ cups whose aerodynamic moment along one turn is characterized by the two more relevant positions with respect to the wind, *α* = 0° and *α* = 180°, see Figure 4. For the equilibrium point, *m*_*A*_ = 0, the rotor can be idealized as a 2-cup rotor with cups, respectively, positioned at the aforementioned angles *α* = 0° (wind pointing to the concave-side of one cup) and *α* = 180° (wind pointing to the convex-side of the other cup). This situation is sketched in Figure 4.

### 1.3. Aim of the Present Work

As explained in the previous subsection, the aim of the present paper is to derive a mathematical model with a strong aerodynamic basis, which could help to explain its behavior as a function of certain parameters. To do so the paper has been organized as follows.  Section 2 describes a mathematical (analytical) model for analyzing cup anemometer behavior. This model is based on the classical 2-cup positions model explained earlier. Although the results from the classical model show some limitations for drag coefficient ratios outside the normal ranges [21] (Brevoort and Joyner [27] measured drag coefficients ratio, *C*_*D*2_/*C*_*D*1_, for different cup shapes in the bracket (0.26, 0.36), whereas Schrenk [28] measured *C*_*D*2_/*C*_*D*1_ = 0.25 for hemispherical cups), it has proven strong enough to provide well-founded explanations of cup anemometer performance [14, 28] and also correlated well with data resulting from the analysis of several commercial cup anemometers [20]. In Section 3, the model is correlated with the results from Table 2. Finally, conclusions are summarized in Section 4.

## 2. Mathematical Model

As stated above, the aim of this paper is to derive a model simple enough to analytically study cup anemometer behavior but also complex enough to include the parameters most relevant to the problem. In this sense, simple classical analytical approximations are unable to reflect the influence of cup size (i.e., cup radius, *R*_*c*_), in the solution for the equilibrium state (*m*_*A*_ = 0; see expressions (12), (13), and (14)) [21, 22]. To overcome this limitation, a nonconstant force distribution on the cup is considered.

The normal-to-the-cup force, *N* (see Figure 4), is generally expressed as

(15) $$\begin{array}{c} N=\frac{1}{2}\rho V_{r}^{2}S_{c}c_{N}, \end{array}$$

where *V*_*r*_ is the relative-to-the-cup wind speed, *S*_*c*_ is the cup's front area, and *c*_*N*_ is the normal-to-the-cup force coefficient (which depends on the angle of *V*_*r*_ with respect to the cup). This force coefficient is normally measured by static wind tunnel testing of an isolated cup, which does not take into account any rotating flow around the cup [21, 22, 27]. Nevertheless, expression (15) is a good approximation for low values of the parameter *r*_*r*_ (i.e., large cup center rotation radius, *R*_*rc*_, in comparison to the cup radius, *R*_*c*_). Some efforts have already been made to include the aforementioned rotating flow effect, considering the dynamic pressure relative to the cup as a function of the distance to the rotation axis [22]. However, the results were not relevant because the normal-to-the-cup aerodynamic coefficient, *c*_*N*_, was still considered constant all over the front surface of the cup, and was based on static wind tunnel measurements. As a result, this paper considers a non-constant pressure distribution on the front area of the cups. The aerodynamic forces on the cup arm are also considered in the mathematical approximation.

The torque on one cup at position *α* = 0° (see Figure 4) is then defined by the following equation:

(16) M1=12ρ[∫Rrc−RcRrc+Rcg1(r)(V−ωr)2l(r)rdr   + Db∫0Rrc−Rc(V−ωr)2rCDbdr],

where *r* is the distance to the rotation axis, *l*(*r*) is the width of the cup at position *r*, *g*_1_(*r*) is the drag coefficient of each section *l*(*r*) of the cup, and *V*,  *ω*,  *R*_*c*_, and *R*_*rc*_ are the wind speed, rotational speed, cup radius, and cup center rotation radius, respectively. Finally, *D*_*b*_ is the diameter of the arm that attaches the cup to the rotor's head and *C*_*Db*_ is the drag coefficient of the cross section of that arm (obviously, referred to the diameter *D*_*b*_). In addition, at position *α* = 180°, the torque on the cup is defined by the following equation:

(17) M2=12ρ[∫Rrc−RcRrc+Rcg2(r)(V+ωr)2l(r)rdr   + Db∫0Rrc−Rc(V+ωr)2rCDbdr],

where *g*_2_(*r*) is the drag coefficient of each section *l*(*r*) of the cup in the new position (note that torques *M*_1_ and *M*_2_ are defined in different rotational directions). For convenience, the new variable *x* = *r* − *R*_*rc*_ is considered hereinafter. If the classical model is considered, the average aerodynamic torque on a cup in a rotor is characterized by the two positions mentioned (*α* = 0° and *α* = 180°). So, as in the equilibrium (autorotation) state, this aerodynamic torque on the rotor must counterbalance the friction torque, *M*_*f*_. That condition can be expressed as

(18) $$\begin{array}{c} N_{c}\left(M_{1}-M_{2}\right)-M_{f}=0, \end{array}$$

where *N*_*c*_ is now a proportional constant that depends on the number of cups. In the classical 2-cup model, expression (11), *N*_*c*_ stands for the exact number of cups, nevertheless, if friction is taken into account and the model is fitted to experimental results without introducing any coefficient to take into account the aerodynamic simplification (*c*_1_ and *c*_2_ in expression (11)), it seems more reasonable to consider the ratio between aerodynamic and friction torques related to the number of cups, and proportional to it in a first approximation. In addition, a linear law is considered for the nonconstant drag coefficients:

(19) $$\begin{array}{c} g_{1}\left(x\right)=C_{d10}\left(1+\delta_{1}\frac{x}{R_{c}}\right);\;\;g_{2}\left(x\right)=C_{d20}\left(1+\delta_{2}\frac{x}{R_{c}}\right), \end{array}$$

where *C*_*d*10_ and *C*_*d*20_ are the average drag coefficients of the cups (*C*_*D*1_ and *C*_*D*2_ in (11) and (12)), *x* is the distance measured from the center of the cup (see Figure 1), and *δ*_1_ and *δ*_2_ are slope constants of the mentioned linear laws. Finally, a friction torque coefficient, *C*_*mf*_, is defined as

(20) $$\begin{array}{c} C_{mf}=\frac{M_{f}}{\left(1/2\right)\rho S_{c}R_{rc}C_{d10}V^{2}N_{c}}. \end{array}$$

Taking into account these last expressions, (18) can be rewritten as

(21) 12ρ∫−RcRcCd10(1+δ1xRc)[V−ω(Rrc+x)]2    ×2Rc1−(xRc)2(Rrc+x)dx −12ρ∫−RcRcCd20(1+δ2xRc)[V−ω(Rrc+x)]2     ×2Rc1−(xRc)2(Rrc+x)dx +12ρDbCDb∫0Rrc−Rc[(V−ωr)2−(V+ωr)2]rdr −12ρScRrcCd10Cmf=0.

If the same linear law is considered for both *g*_1_(*x*) and *g*_2_(*x*); that is,

(22) $$\begin{array}{c} \delta_{1}=\delta_{2}=\delta , \end{array}$$

and coefficient *k*_*D*_ is now defined as

(23) $$\begin{array}{c} k_{D}=\sqrt{\frac{C_{d20}}{C_{d10}}}, \end{array}$$

then the following expression can be derived from (21):

(24) (1−kD2)(1+δ4rr−Cmf1−kD2)−2Ω(1+kD2) ×(1+δ2rr+14rr2+ε1+kD2)+Ω2(1−kD2) ×(1+34δrr+34rr2+18rr3)=0,

where

(25) $$\begin{array}{c} \varepsilon =\frac{2}{3\pi }\frac{D_{b}}{R_{rc}}\frac{C_{Db}}{C_{d10}}\frac{1}{r_{r}^{2}}{\left(1-r_{r}\right)}^{3}=\varepsilon_{r}\frac{1}{r_{r}^{2}}{\left(1-r_{r}\right)}^{3}. \end{array}$$

As previously indicated, *r*_*r*_ = *R*_*c*_/*R*_*rc*_ is the ratio of cup radius to cup center rotation radius in the equations above.

Finally, a simple expression can be obtained:

(26) $$\begin{array}{c} \Omega^{2}-2\Omega \gamma \left(1+a\right)+\left(1+b\right)=0, \end{array}$$

where

(27) γ=1+kD21−kD2,a=1+a11+a0−1=a1+a01+a0,b=1+a21+a0−1=a2+a01+a0,a0=34δrr+34rr2+18rr3,a1=δ2rr+14rr2+ε1+kD2,a2=δ4rr−Cmf1+kD2.

It must also be said that expression (26) is dependent on coefficients (*a*_0_,  *a*_1_,  *a*_2_,  *a*, and  *b*), which are low when compared to 1. This makes it possible to derive solutions using Taylor series expansions. Also, if friction is considered negligible (*C*_*mf*_ = 0), the drag coefficient distributions along the cups are considered constant (*δ* = 0), and no aerodynamic drag is considered on the cup arms (*C*_*Db*_ = 0); expression (26) turns into

(28) $$\begin{array}{c} \Omega^{2}-2\Omega \gamma +1=0, \end{array}$$

and the solutions are

(29) $$\begin{array}{c} \Omega =+\gamma \pm \sqrt{\gamma^{2}-1}. \end{array}$$

Obviously, the only logical solution is the one with a negative sign (Ω < 1):

(30) $$\begin{array}{c} \Omega =\Omega_{0}=\frac{1-k_{D}}{1+k_{D}}. \end{array}$$

As stated in Section 1, this is the solution for the simpler 2-cup modeling of cup anemometer behavior.

Going back to (26), if small perturbations are considered (drag distribution on cups, friction, etc.), it is possible to find an approximate asymptotic solution:

(31) $$\begin{array}{c} \Omega =\Omega_{0}+\Omega_{1}, \end{array}$$

where Ω_1_ ≪ Ω_0_. In addition, considering *a*, *b* ≪ 1 as stated above, the following expression can be derived (see Appendix B):

(32) Ω1=Ω0aγ−b/2Ω0−γ=(1+kD2(1+kD)2(−14δrr+ε1+kD2)  −12(−δ2rr−Cmf1−kD2))×(−2kD1−kD2)−1≅−δrr4Ω0−12kDΩ0ε−14CmfkD.

Finally, (31) can be expressed as

(33) $$\begin{array}{c} \Omega =\Omega_{0}-\left[\frac{\delta r_{r}}{4}\Omega_{0}+\frac{1}{2k_{D}}\Omega_{0}\varepsilon +\frac{1}{4}\frac{C_{mf}}{k_{D}}\right]. \end{array}$$

**Figure 5:**
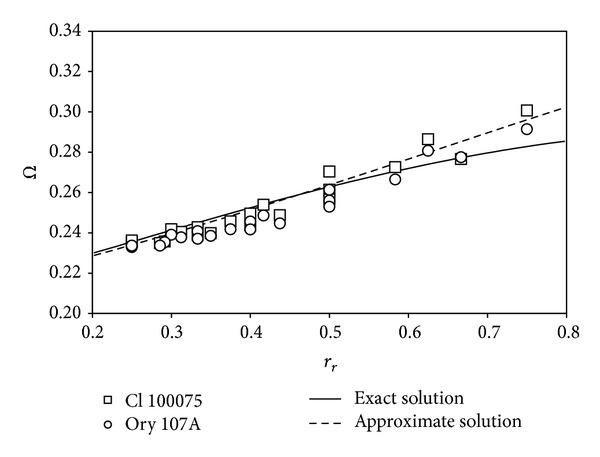
Parameter Ω estimated with the results from the calibrations performed on the Climatronics 100075 and Ornytion 107A anemometers, Ω = 1/*K*_*S*_ (see also Figure 2). The graph also includes the results from the exact and approximate solutions for the proposed analytical model ((24) and (33), resp.), fitted to the experimental data.

In Figure 5, the exact and approximate solutions for the proposed method ((24) and (33), resp.) are fitted to the experimental results (Table 2), without taking into account the offset of the transfer function; that is, Ω = 1/*K*_*S*_ (see expression (9)). These solutions, *k*_*D*_ = 0.656 and *δ* = −1.8, were calculated without considering the effect of the rotor arm or friction (*ε* = 0 and *C*_*mf*_ = 0). As shown in the figure, there is good agreement between the solutions. Several conclusions can be derived from (32). As expected, the contribution of the rotor arm and friction, *ε* and *C*_*mf*_, tends to reduce the steady rotational speed, Ω. On the other hand, the fitting to the experimental results reveals a negative value of *δ*, indicating a higher average aerodynamic load on the closest-to-the-rotating-axis area of the cup. This is a rather surprising result, which could be produced by both unsteady and rotational aerodynamic effects.

In order to analyze anemometer transfer function (3) using the model developed to introduce the effects of the rotor arms, friction, and the aforementioned “inverse” distribution of the aerodynamic load on the cup, the anemometer factor, *K*, must be expressed as a function of 1/Ω:

(34) VωRrc=1Ω=1Ω0+Ω1≅1Ω011+Ω1/Ω0≅1Ω0(1−Ω1Ω0)=1Ω0−1Ω02Ω1=1Ω0+1Ω02 ×[δrr4Ω0+12kDΩ0ε+14kDCmf]=1Ω0(1+14δrr+12kDε+14kDCmfΩ0),

and then

(35) V=ωRrc1Ω=ωRrc1Ω0(1+14δrr+12kDε+14kDΩ0Cmf)=ωRrc1Ω0[1+14δrr+12kDεr1rr2(1−rr)3] +14kDΩ02ωRrcCmf.

The first term in the above equation directly affects the first term in transfer function (3), *A*_*r*_ *ω*, and can be divided into three other terms. The first,

(36) $$\begin{array}{c} \omega R_{rc}\frac{1}{\Omega_{0}}, \end{array}$$

only depends on the cup aerodynamics and not on the slight effects considered. The second one,

(37) $$\begin{array}{c} \omega R_{rc}\frac{1}{\Omega_{0}}\frac{1}{4}\delta r_{r}, \end{array}$$

implies a correction of the slope, linear with *r*_*r*_, that would be included in the second term in expression (10), respecting the negative sign as *δ* < 0. Finally, the third term,

(38) $$\begin{array}{c} \omega R_{rc}\frac{1}{\Omega_{0}}\frac{1}{2k_{D}}\varepsilon_{r}\left(\frac{1}{r_{r}^{2}}-\frac{3}{r_{r}}+3-r_{r}\right), \end{array}$$

can also be separated into three different contributions to the slope of the transfer function:

(1) one correction not dependent on *r*_*r*_
  (39) $$\begin{array}{c} \left(\frac{3}{2}\right)\frac{\varepsilon_{r}}{k_{D}}, \end{array}$$
(2) one correction linear with *r*_*r*_, which also respects the negative sign in the second term in expression (10)
  (40) $$\begin{array}{c} -\left(\frac{1}{2}\right)\frac{\varepsilon_{r}}{k_{D}}r_{r}, \end{array}$$
(3) and, finally, a nonlinear term
  (41) $$\begin{array}{c} \left(\frac{1}{2}\right)\frac{\varepsilon_{r}}{k_{D}}\left(\frac{1}{r_{r}^{2}}-\frac{3}{r_{r}}\right). \end{array}$$

With regard to the second term in (35), it represents a friction velocity term, *V*_*f*_, which can be expressed as

(42) Vf=ωRrc4kDΩ02(Mf(1/2)ρScRrCd10V2)=14kDΩ0Mf(1/2)ρScRrCd10V.

If the second-order polynomial approximation for the friction torque mentioned in Section 1 is considered:

(43) $$\begin{array}{c} M_{f}=M_{f0}+M_{f1}\omega +M_{f2}\omega^{2}, \end{array}$$

then (42) turns into

(44) $$\begin{array}{c} V_{f}=\frac{1}{4k_{D}\rho S_{c}C_{d10}}\left(\frac{M_{f0}}{\omega R_{rc}^{2}}+\frac{M_{f1}}{R_{rc}^{2}}+\frac{M_{f2}}{R_{rc}^{2}}\omega \right), \end{array}$$

where the first term,

(45) $$\begin{array}{c} \frac{M_{f0}}{\omega R_{rc}^{2}}, \end{array}$$

indicates the deviation from the ideal velocity (36) at low rotational speeds. This effect is mentioned in Section 1, where the non-linear behavior of anemometer constant *K* at low wind speeds is described. The second term,

(46) $$\begin{array}{c} \frac{M_{f1}}{R_{rc}^{2}}, \end{array}$$

involves a contribution that does not depend on *ω*, so it represents the offset constant, *B*, in transfer function (3). Finally, the third term,

(47) $$\begin{array}{c} \frac{M_{f2}}{R_{rc}^{2}}\omega , \end{array}$$

represents a contribution to transfer function slope *A*_*r*_.

From expression (42), it can also be assumed that the friction term, *V*_*f*_, is inversely proportional to the cup surface, *S*_*c*_, meaning that bigger cups would be translated into a lower friction term. Nevertheless, it must be taken into account that bigger cups also experience greater lateral forces on the anemometer rotor shaft, increasing the friction.

As mentioned, the results included in Table 2 summarize the results from [21]. Bearing in mind these results (see expressions (3) to (7)), it is possible to derive the following equation:

(48) V−B=(dArdRrRr+Ar0)f=2πKARrcf+2πKcRcf=KARrcω+KcRcω,

where

(49) $$\begin{array}{c} K_{A}=\frac{1}{2\pi }\frac{\text{d}A_{r}}{\text{d}R_{rc}} \end{array}$$

is the contribution to the anemometer factor that depends on the cup center rotation radius, *R*_*rc*_, and:

(50) $$\begin{array}{c} K_{c}=\frac{1}{2\pi R_{c}}A_{r0}, \end{array}$$

is the contribution to the anemometer factor that does not depend on the cup center rotation radius but on the cup radius, *R*_*c*_. Both coefficients, *K*_*A*_ and *K*_*c*_, are dimensionless and can be related to the anemometer factor defined in expression (9) through the following equation:

(51) $$\begin{array}{c} K_{S}=K_{A}+K_{c}r_{r}. \end{array}$$

If we compare expression (48) to the same expression obtained from the theoretical model (35), neglecting the effect of rotor arms or friction (*ε* = *C*_*mf*_ = 0), that is,

(52) $$\begin{array}{c} V=\frac{\omega }{\Omega_{0}}R_{rc}\left(1+\frac{1}{4}\delta r_{r}\right)=\frac{\omega R_{rc}}{\Omega_{0}}+\frac{\omega R_{c}}{\Omega_{0}}\frac{\delta }{4}, \end{array}$$

the two terms of the aforementioned (48) can be explained from a theoretical basis. First, the slope of the anemometer calibration curve (a transfer function based on the rotation frequency; that is, expression (3) instead of expression (2)), has one term, *K*_*A*_ *R*_*rc*_ *ω*, proportional to the cup center rotation radius, *R*_*rc*_, with the proportionality constant *K*_*A*_ = 1/Ω_0_. Second, the last term of the aforementioned anemometer transfer function slope, *K*_*c*_ *R*_*c*_ *ω*, which does not depend on *R*_*rc*_, depends on both the load (pressure) distribution along the cups, *δ*, and the cup radius, *R*_*c*_. It should be underlined that this second term in the equation of the anemometer factor only appears in the theoretical model if the “inverse” force distribution along the cup is considered.

Comparing expressions (51) and (52) leads to

(53) $$\begin{array}{c} \frac{1}{4}\frac{\delta }{\Omega_{0}}≅K_{c}=\frac{A_{r0}}{2\pi R_{c}}. \end{array}$$

Finally, combining expressions (49) and (53) with the above equation it is possible to rewrite expression (51) in terms of the mentioned ratio of cup radius to cup center rotation radius, *r*_*r*_ = *R*_*c*_/*R*_*rc*_, as

(54) $$\begin{array}{c} K_{S}=\frac{1}{2\pi }\left[\frac{\text{d}A_{r}}{\text{d}R_{rc}}+\frac{\pi }{2}\frac{\delta }{\Omega_{0}}r_{r}\right], \end{array}$$

which, taking into account the negative value of coefficient *δ*, results in an equation very similar to the one derived from the experimental results (10). Nevertheless, it must also be noted that both terms in expression (10) include the slight effects of rotor arms and friction, which are not considered in the above equation.

## 3. Results and Discussion

**Figure 6:**
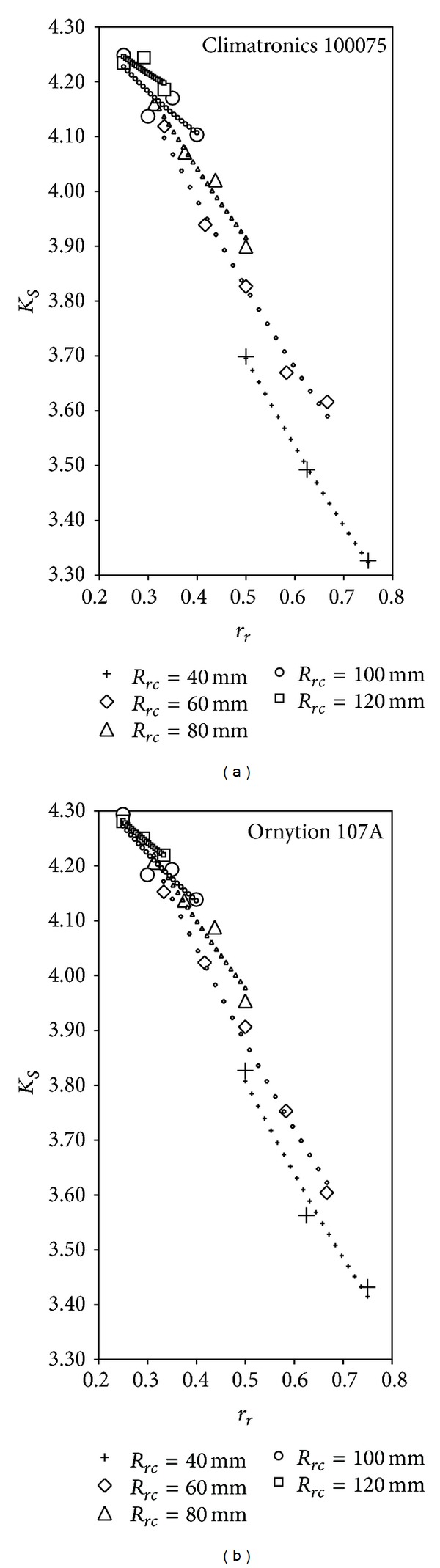
Anemometer factor, *K*_*S*_, from the calibrations performed on the Climatronics 100075 and Ornytion 107A as a function of parameter *r*_*r*_, for rotors with the same cup center rotation radius, *R*_*rc*_. The small symbols correspond to the analytical model (expression (24)), fitted to the experimental results. See also Table 3.

In Figure 6, anemometer factor *K*_*S*_, obtained from the calibrations performed on the anemometers used in the testing campaign, is shown as a function of the parameter *r*_*r*_, for rotors with the same cup center rotation radius, *R*_*rc*_. The figure also compares these experimental results to those from the proposed analytical model, without taking into account the effect of rotor arms or friction (*ε* = *C*_*mf*_ = 0). In each case, the complete expression from the analytical model (24) is fitted by adjusting both *k*_*D*_ and *δ*.  Table 3 includes the values of these parameters for each case, along with the slope, d*K*_*S*_/d*r*_*r*_, and offset, *K*_*S*0_, of the linear fittings and the corresponding determination coefficient, *R* ^2^. Obviously, d*K*_*S*_/d*r*_*r*_ = *K*_*c*_ and *K*_*S*0_ = *K*_*A*_, from expression (51).

Good agreement between the proposed model and the testing results is observed in Figure 6; in other words, the effect of parameter *r*_*r*_ is correctly reflected by the model. However, it is also fair to say that greater differences between the model and the experimental results are observed in the figure for higher values of *R*_*rc*_. This dispersion of the testing results could be explained by the fact that a longer cup center rotation radius, *R*_*rc*_, lowers the rotation speed, making the rotation movement less constant and more influenced by the third harmonic term [4, 136] (the rotation speed of a cup anemometer can be decomposed into different harmonic terms using the Fourier expansion: *ω*(*t*) = *ω*_0_ + *ω*_1_sin(*ω*_0_ *t* + *φ*_1_) + *ω*_2_sin(2*ω*_0_ *t* + *φ*_2_) + *ω*_3_sin⁡(3*ω*_0_ *t* + *φ*_3_)…. Leaving aside the average term, *ω*_0_, the third harmonic term, *ω*_3_, is the most important term of the expansion in normal circumstances due to the 3-cup rotor shape).

**Figure 7:**
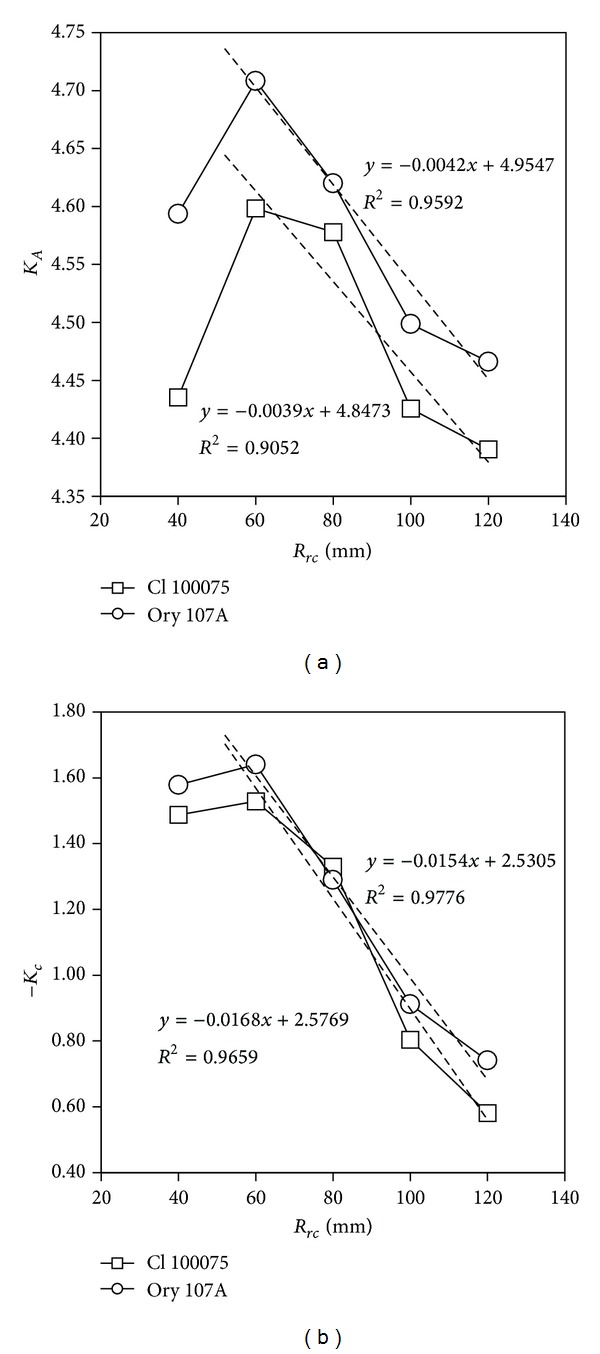
Coefficients *K*_*A*_ and *K*_*c*_, plotted as a function of *R*_*rc*_, for both anemometers, Climatronics 100075 and Ornytion 107A. The linear fittings to the data have been included in the graphs for *R*_*rc*_ ≥ 60 mm (in other words, they were calculated excluding the point *R*_*rc*_ = 40 mm).

To return to the linear fittings in Table 3, taking into account the high values of determination coefficient *R* ^2^, it seems that the linear approximation previously derived (51) reflects the anemometer behavior quite well. It is also possible to observe the variation of both coefficients, *K*_*A*_ and *K*_*c*_, as a function of *R*_*rc*_, which is plotted in Figure 7. For both anemometers, Climatronics 100075 and Ornytion 107A, these coefficients show approximate linear behavior for *R*_*rc*_ > 60 mm:

(55) $$\begin{array}{c} K_{A}=K_{A0}+K_{AS}R_{rc},\\ K_{c}=K_{c0}+K_{cS}R_{rc}. \end{array}$$

For lower cup center rotation radius values, *R*_*rc*_ < 60 mm, the tendency of both coefficients, *K*_*A*_ and *K*_*c*_, to decrease the anemometer factor as a function of *R*_*rc*_ is alleviated, probably due to cup-wake interaction.  Table 4 includes the coefficients of the aforementioned linear fitting to *K*_*A*_ and *K*_*c*_ for *R*_*rc*_ > 60 mm (expressions (55)). The following conclusions can be reached from these fittings.

- The behavior of coefficient *K*_*c*_ is very similar for both anemometers, an average expression being: *K*_*c*_ = −2.55 + 0.0161*R*_*rc*_. (*R*_*rc*_ expressed in mm). This parameter seems to depend only on the rotor shape (cups size and rotor diameter) and not on the anemometer shape (anemometer body).
- The linear fittings to parameter *K*_*A*_ show similar values for the slope, *K*_*AS*_, but different values for the offset, *K*_*A*0_. This difference could be explained by different friction on the shaft/bearings system or a different anemometer shape (“neck” thickness).

From expressions (51) and (55) it is possible to obtain, in combination with the anemometer factor equation,

(56) $$\begin{array}{c} K_{S}=\frac{V-B}{\omega R_{rc}}=\left(K_{A0}+K_{AS}R_{rc}\right)+\left(K_{c0}+K_{cS}R_{rc}\right)r_{r}, \end{array}$$

a new expression for wind speed,

(57) $$\begin{array}{c} V=\left[K_{A0}+K_{AS}R_{rc}+K_{cS}R_{c}\right]R_{rc}\omega +K_{c0}R_{c}\omega +B, \end{array}$$

that is, an expression indicating a term related to the anemometer transfer function slope, *K*_*c*0_ *R*_*c*_, which does not depend on *R*_*rc*_. The effect of this term was experimentally observed [21]; see expression (6).

To return to (53), it is possible to estimate the gradient along the cup of the normal-to-the-cup aerodynamic force, *δ* (see expressions (19) and (22)),

(58) $$\begin{array}{c} \delta ≅\frac{2}{\pi }\Omega_{0}\frac{A_{r0}}{R_{c}}. \end{array}$$

**Figure 8:**
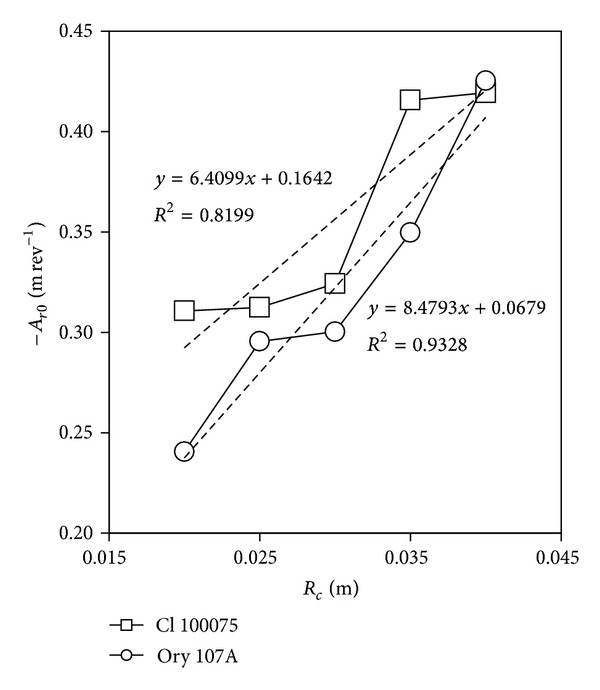
Constant term of the slope for calibration coefficient *A*_*r*0_ (see expression (4)) from the testing results, as a function of anemometer cup radius, *R*_*c*_. Linear fittings have been added to the graph.

Figure 8 shows coefficient *A*_*r*0_ as a function of the anemometer cup radius, *R*_*c*_. Considering an average value between *A*_*r*0_/*R*_*c*_ = −12.2 (Climatronics 100075) and *A*_*r*0_/*R*_*c*_ = −10.9 (Ornytion 107A), the above equation can be rewritten as

(59) $$\begin{array}{c} \delta ≅-7.38\frac{1-k_{D}}{1+k_{D}}. \end{array}$$

The above equation clearly indicates the aforementioned “inverse” aerodynamic load distribution on the anemometer cups. In other words, the aerodynamic load on the rotating cup is higher in the area closest to the rotating axis. This effect, which could be attributed to local changes in wind direction along the cup, to the effect of rotating flow on the pressure distribution along the cup, or to wake interaction, should not be neglected in the development of new models for studying cup anemometers.

**Figure 9:**
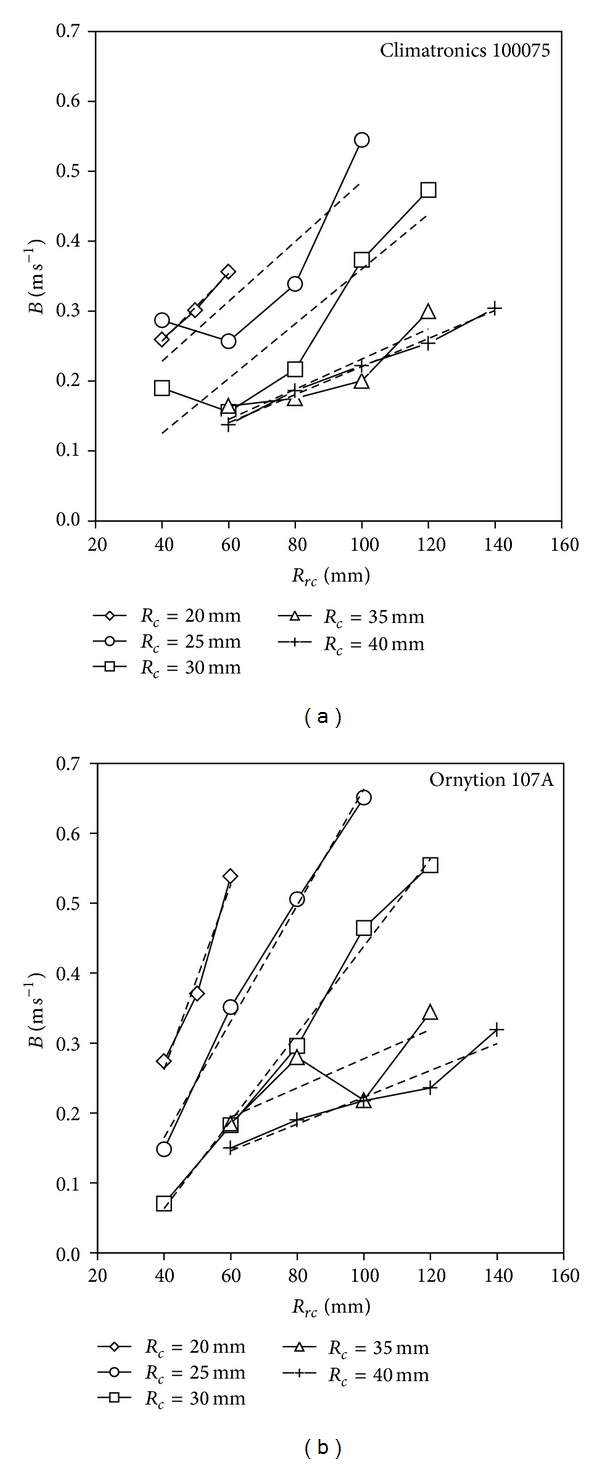
Coefficient *B* for the calibrations performed (see Table 2) on the Climatronics 100075 and the Ornytion 107A anemometers, shown as a function of the cup center rotation radius, *R*_*rc*_, for each cup radius, *R*_*c*_. Linear fittings to the data are also shown in the graphs (the coefficients of these fittings, d*B*/d*R*_*rc*_ and *B*_0_, are included in Table 2).

In a previous paper [21], the offset of the transfer function (coefficient *B* in expressions (2) and (3)) was experimentally fitted to the expression, depending on the cup center rotation radius, *R*_*rc*_, and the front area of the cups, *S*_*c*_ (see (7)). Taking that expression into account, offset *B* depends linearly on *R*_*rc*_.  Figure 9 shows coefficient *B* for the calibrations performed (see Table 2) as a function of the cup center rotation radius, *R*_*rc*_, for each cup radius, *R*_*c*_. Linear fittings to the data are also shown in the graphs (the coefficients for these fittings, d*B*/d*R*_*rc*_ and *B*_0_, are included in Table 2). The linear trend mentioned earlier is shown in the graphs, although it is much clearer in the case of the Ornytion 107A. This could be explained by the different effects of aging or wear and tear, on both anemometers. In the testing campaign considered in this paper, the Ornytion 107A was new, whereas the Climatronics 100075 had been used for internal procedures at the IDR for several years (some degree of degradation regarding this anemometer was previously illustrated in [82]). Another effect observed in the curves corresponding to *R*_*c*_ = 25 and *R*_*c*_ = 30 for the Climatronics 100075 is a deviation from the linear trend for lower values of *R*_*rc*_. This suggests an interaction between the cup and the wake generated at the anemometer's “neck.” This effect is not observed in the graph for the Ornytion 107A, probably because this anemometer has a thinner “neck,” which also produces a thinner wake. Finally, a decrease in the slope, d*B*/d*R*_*rc*_, along with the cup radius, *R*_*c*_, can be observed in Figure 9.

## 4. Conclusions

In this study, cup anemometer response was analyzed using the 2-cup analytical model. The model was fitted to experimental data from 42 calibrations performed on two different cup anemometers (Climatronics 10075 and Ornytion 107A), equipped with 21 different cup rotors (conical cups, with varying cup sizes and distances to the rotation axis). The major conclusions resulting from this study are as follows.

- The anemometer factor, *K*_*S*_, for cup anemometers equipped with conical cups depends linearly on the shape parameter *r*_*r*_ (the ratio of the cup radius to the cup center rotation radius, *r*_*r*_ = *R*_*c*_/*R*_*rc*_), *K*_*S*_ = *K*_*A*_ + *K*_*c*_ *r*_*r*_, within the range studied (from *r*_*r*_ = 0.25 to *r*_*r*_ = 0.75). The slope of this linear behavior, *K*_*c*_, seems to depend only on the rotor shape but not on the anemometer body (for the anemometers tested).
- The results indicate that the aerodynamic force distribution along the rotating cups is not uniform, as the calculated aerodynamic force is higher closer to the rotating axis, which explains the linear correction term *K*_*c*_ *r*_*r*_ to the anemometer factor.
- The classical 2-cup analytical model modified with the nonconstant force distribution along the rotating cup of the anemometer seems to be accurate enough for studying the complex aerodynamics effects involved in the rotor performance.

## Figures and Tables

**Figure 10:**
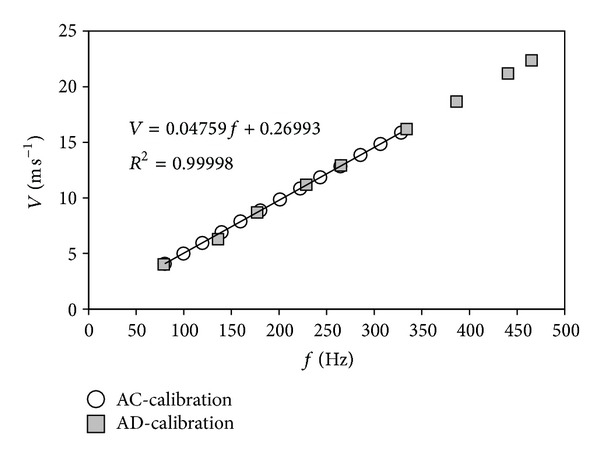
Results from two calibrations performed at the S4 anemometer calibration wind tunnel of the IDR/UPM Institute, on the same cup anemometer (Thies Clima 4.3350) following two different procedures, AC and AD. AC calibrations follow strictly MEASNET procedure (wind speeds ranging from 4 m s^−1^ to 16 m s^−1^, and 13 measurement points are taken), whereas AD calibrations are carried out over a broader wind speed range (from 4 m s^−1^ to 23 m s^−1^) and less measurement points are taken (9 instead of 13).

**Table 1:** Research carried out on cup anemometer behavior/performances. References classified by areas of study and research. Some references regarding applications have also been included.

Research	Mathematical model	Experimental research
Static and dynamic response		
Aerodynamic force on the cups; shape of the anemometer	(i) Schrenk 1929 [28] (ii) Ramachandran 1969 [23] (iii) Kondo et al. 1971 [24] (iv) Dahlberg et al. 2001 [8] (v) Winkel et al. 2007 [29] (vi) Pindado et al. 2012 [21] (vii) Pindado et al. 2013 [22] (viii) Potsdam et al. 2013 [30]	(i) Patterson 1926 [31] (ii) Schrenk 1929 [28] (iii) Pinkerton 1930 [32] (iv) Hubbard and Brescoll 1934 [33] (v) Brevoort and Joyner 1935 [27] (vi) Fergusson 1939 [34] (vii) Albright and Klein 1941 [35] (viii) Fritschen 1967 [36] (ix) Ramachandran 1969 [23] (x) Kondo et al. 1971 [24] (xi) Lindley 1975 [37] (xii) Lockhart 1985 [38] (xiii) Pedersen and Paulsen 1999 [39] (xiv) Dahlberg et al. 2001 [8] (xv) Hunter et al. 2003 [40] (xvi) Winkel et al. 2007 [29] (xvii) Pindado et al. 2011 [20] (xviii) Pindado et al. 2012 [21] (xix) Pindado et al. 2013 [22] (xx) Pindado et al. 2013 [4]

Average rotational speed estimation; vertical component of the wind speed; overspeeding. turbulence	(i) Schrenk 1929 [28] (ii) Scrase and Sheppard 1944 [41] (iii) Sanuki 1952 [42] (iv) Ramachandran 1969 [23] (v) Ramachandran 1969 [43] (vi) Torochkov and Surazhskiy 1969 [44] (vii) Acheson 1970 [45] (viii) Kondo et al. 1971 [24] (ix) Hyson 1972 [15] (x) Wyngaard et al. 1974 [13] (xi) Kaganov and Yaglom 1976 [11] (xii) Busch and Kristensen 1976 [18] (xiii) Busch et al. 1980 [46] (xiv) Wyngaard 1981 [14] (xv) Coppin 1982 [16] (xvi) Hayashi 1987 [47] (xvii) Frenzen 1988 [48] (xviii) Snow et al. 1989 [49] (xix) Chang and Frenzen 1990 [17] (xx) Hunter 1990 [50] (xxi) Skarstein et al. 1992 [51] (xxii) Kristensen 1993 [52] (xxiii) Kristensen 1994 [53] (xxiv) Fabian 1995 [54] (xxv) Westermann 1996 [55] (xxvi) Kristensen 1998 [19] (xxvii) Hristov et al. 2000 [56] (xxviii) Kristensen 2000 [57] (xxix) Dahlberg et al. 2001 [8] (xxx) Kristensen 2002 [3] (xxxi) Kristensen and Hansen 2002 [58] (xxxii) Pedersen 2003 [10] (xxxiii) Kristensen et al. 2003 [59] (xxxiv) Pedersen 2004 [60] (xxxv) Solov'ev et al. 2004 [12] (xxxvi) Yahaya and Frangi 2004 [61] (xxxvii) Kristensen and Hansen 2005 [62] (xxxviii) Dahlberg et al. 2006 [9] (xxxix) Bégin-Drolet et al. 2011 [63] (xl) Pindado et al. 2012 [21] (xli) Bégin-Drolet et al. 2013 [64]	(i) Brazier 1921 [65] (ii) Schrenk 1929 [28] (iii) Marvin 1932 [66] (iv) Marvin 1934 [67] (v) Spilhaus and Rossby 1934 [68] (vi) Brevoort and Joyner 1935 [27] (vii) Fergusson 1939 [34] (viii) Scrase and Sheppard 1944 [41] (ix) Deacon 1951 [69] (x) MacCready 1966 [70] (xi) Bernstein 1967 [71] (xii) Izumi and Barad 1970 [72] (xiii) Camp et al. 1970 [73] (xiv) Kondo et al. 1971 [24] (xv) Hyson 1972 [15] (xvi) Lindley and Bowen 1974 [74] (xvii) Wyngaard et al. 1974 [13] (xviii) Lindley 1975 [37] (xix) Baynton 1976 [75] (xx) Busch et al. 1980 [46] (xxi) Coppin 1982 [16] (xxii) Hayashi 1987 [47] (xxiii) Hunter 1990 [50] (xxiv) Morris et al. 1992 [76] (xxv) Fabian 1995 [54] (xxvi) Westermann 1996 [55] (xxvii) Kristensen 2000 [57] (xxviii) Dahlberg et al. 2001 [8] (xxix) Pedersen 2003 [10] (xxx) Hunter et al. 2003 [40] (xxxi) Pedersen 2004 [60] (xxxii) Solov'ev et al. 2004 [12] (xxxiii) Pedersen and Paulsen 1999 [39] (xxxiv) Kristensen 2002 [58] (xxxv) Yahaya and Frangi 2004 [61] (xxxvi) Dahlberg et al. 2006 [9] (xxxvii) Pedersen et al. 2006 [77] (xxxviii) Bégin-Drolet et al. 2011 [63] (xxxix) Pindado et al. 2012 [21] (xl) Bégin-Drolet et al. 2013 [64] (xli) Pindado et al. 2013 [22] (xlii) Pindado et al. 2013 [4]

Effect of bearings system; friction.	(i) Fabian 1995 [54] (ii) Pedersen and Paulsen 1999 [39] (iii) Dahlberg et al. 2001 [8] (iv) Pedersen 2003 [10] (v) Pedersen 2004 [60] (vi) Dahlberg et al. 2006 [9]	(i) Baynton 1976 [75] (ii) Fabian 1995 [54] (iii) Pedersen and Paulsen 1999 [39] (iv) Dahlberg et al. 2001 [8] (v) Pedersen 2003 [10] (vi) Pedersen 2004 [60] (vii) Dahlberg et al. 2006 [9] (viii) Pedersen et al. 2006 [77]

Performance degradation	(i) Siegel and Lee 2011 [78] (ii) Cassity et al. 2012 [79] (iii) Sun et al. 2012 [80]	(i) Zlatanovic and Zlatanovic 2012 [81] (ii) Pindado et al. 2012 [82]

Output signal post-processing		
Sampling; filtering; correction of the measured velocity	(i) Wieringa 1980 [83] (ii) Wyngaard et al. 1982 [84] (iii) Moore 1986 [85] (iv) Hayashi 1987 [47] (v) Skarstein et al. 1992 [51] (vi) Ebert et al. 1995 [86] (vii) Barnard et al. 1998 [87] (viii) Hristov et al. 2000 [56] (ix) Kristensen et al. 2003 [59] (x) Selyaninov 2004 [88] (xi) Solov'ev et al. 2004 [12] (xii) Yahaya and Frangi 2004 [61] (xiii) Siegel and Lee 2011 [78] (xiv) Bégin-Drolet et al. 2013 [64]	(i) Bernstein 1967 [71] (ii) Wieringa 1980 [83] (iii) Wyngaard et al. 1982 [84] (iv) Hayashi 1987 [47] (v) Ebert et al. 1995 [86] (vi) Solov'ev et al. 2004 [12] (vii) Yahaya and Frangi 2004 [61] (viii) Bégin-Drolet et al. 2013 [64]

Performance on the field		
Climatic conditions (change of air density, rain, ice, extreme weather conditions, etc.)	(i) Dentler 1978 [89] (ii) Fortin et al. 2005 [90]	(i) Gates and Thompson 1986 [91] (ii) Kimura et al. 2001 [92] (iii) Makkonen et al. [93] (iv) Fortin et al. 2005 [90] (v) Pindado et al. 2012 [94] (vi) Hobby et al. 2013 [95]

Anemometer allocation on towers	(i) Wieringa 1980 [83] (ii) Wyngaard 1981 [96] (iii) Wyngaard et al. 1982 [84] (iv) Hansen and Pedersen 1999 [97] (v) Lubitz 2009 [98]	(i) Izumi and Barad 1970 [72] (ii) Burt 1975 [99] (iii) Wieringa 1980 [83] (iv) Wyngaard et al. 1982 [84] (v) Morris et al. 1992 [76] (vi) Pedersen et al. 1992 [100] (vii) Hunter et al. 2003 [40] (viii) Orlando et al. 2011 [101] (ix) Farrugia and Sant 2013 [102]

Performance on the field; recalibration on the field; comparison with Lidar and Sodar (and other instruments)	(i) Kristensen et al. 1991 [103] (ii) Siegel and Lee 2011 [78] (iii) Cassity et al. 2012 [79] (iv) Sun et al. 2012 [80]	(i) Camp 1966 [104] (ii) Kristensen et al. 1991 [103] (iii) Petersen et al. 1998 [105] (iv) Albers et al. 2000 [7] (v) Albers and Klug 2001 [6] (vi) Papadopoulos et al. 2001 [106] (vii) Hunter et al. 2001 [107] (viii) Paulsen et al. 2007 [108] (ix) Wagner et al. 2011 [109] (x) Lang and McKeogh 2011 [110] (xi) Bradley 2013 [111] (xii) Hasager et al. 2013 [112] (xiii) Sanz Rodrigo et al. 2013 [113]

Wind tunnel testing and calibration		
Description; procedure; uncertainties	(i) Robinson 1878 [114] (ii) Robinson 1880 [115] (iii) Brazier 1920 [116] (iv) Hunter 1990 [50] (v) López Peña and Duro 2003 [117] (vi) Eecen and De Noord 2005 [118] (vii) Coquilla et al. 2007 [2] (viii) Piccato et al. 2010 [119] (ix) Piccato et al. 2011 [120] (x) Coquilla 2012 [121]	(i) Robinson 1878 [114] (ii) Robinson 1880 [115] (iii) Brazier 1920 [116] (iv) Camp 1966 [104] (v) Baynton 1976 [75] (vi) Lockhart 1985 [38] (vii) Lockhart 1987 [122] (viii) Gates and Thompson 1986 [91] (ix) Hunter 1990 [50] (x) Makkonen and Helle 1994 [123] (xi) Fabian 1995 [54] (xii) MEASNET 1997 [26] (xiii) Pedersen and Paulsen 1999 [39] (xiv) Hunter et al. 2001 [107] (xv) ASTM International 2002 [124] (xvi) Hunter et al. 2003 [40] (xvii) Eecen and De Noord 2005 [118] (xviii) International Electrotechnical Commision 2005 [5] (xix) Dahlberg 2006 [125] (xx) Coquilla et al. 2007 [2] (xxi) Coquilla and Obermeier 2008 [126] (xxii) MEASNET 2009 [25] (xxiii) Piccato et al. 2010 [119] (xxiv) Pindado et al. 2011 [20] (xxv) Piccato et al. 2011 [120] (xxvi) Westermann et al. 2011 [127] (xxvii) Hansen et al. 2012 [128] (xxviii) Coquilla 2012 [121] (xxix) Gkanias and Katsanevakis 2012 [129]

Instrumentation	(i) McBean 1972 [130] (ii) Sheppard et al. 1972 [131] (iii) Lockhart 1985 [38] (iv) Moore 1986 [85]	(i) Sheppard et al. 1972 [131] (ii) Hobby et al. 2013 [95]

Design and performance		
Design; anemometer classification	(i) Pedersen and Paulsen 1999 [39] (ii) Kristensen 2002 [58] (iii) Pedersen 2003 [10] (iv) Pedersen 2004 [60] (v) Dahlberg et al. 2006 [9] (vi) Pindado et al. 2013 [22]	(i) Frenzen 1968 [132] (ii) Wellman 1968 [133] (iii) Mazzarella 1972 [134] (iv) Lindley 1975 [37] (v) Pedersen and Paulsen 1999 [39] (vi) Dahlberg et al. 2001 [8] (vii) Kristensen 2002 [58] (viii) Pedersen 2003 [10] (ix) Pedersen 2004 [60] (x) Dahlberg et al. 2006 [9] (xi) Pedersen et al. 2006 [77] (xii) Choon et al. 2012 [135] (xiii) Pindado et al. 2013 [22]

**Table 2:** Results of the calibrations performed on the Climatronics 100075 and Ornytion 107A anemometers from [21]. The calibration constants, *A*, *B*, and *A*_*r*_, are indicated together with the simplified anemometer factor, *K*_*S*_, and some geometric characteristics of each rotor tested (cup radius, *R*_*c*_, cup center rotation radius, *R*_*rc*_, and the ratio between them, *r*_*r*_). The coefficients of the linear fittings to the calibration constants, d*A*_*r*_/d*R*_*rc*_, *A*_*r*0_, d*B*/d*R*_*rc*_, and *B*_0_, are also included in the table; see expressions (4) and (5) in the text.

Rotor	*R*_*c*_ [mm]	*R*_*rc*_ [mm]	*r*_*r*_ (*R*_*c*_/*R*_*rc*_)	*A* [m pulse^−1^]	*B* [m s^−1^]	*A*_*r*_ [m rev^−1^]	*K*_*S*_	d*A*_*r*_/d*R*_*rc*_ [rev^−1^]	*A*_*r*0_ [m rev^−1^]	d*B*/d*R*_*rc*_ [s^−1^]	*B*_0_ [m s^−1^]
Climatronics 100075											

20/40	40	40	0.5000	0.0310	0.2593	0.9295	3.70	31.16	−3.107 10^−1^	4.845	6.327 10^−2^
20/50	40	50	0.4000	0.0420	0.3010	1.2592	4.01				
20/60	40	60	0.6667	0.0518	0.3562	1.5526	4.12				

25/40	50	40	0.6250	0.0293	0.2867	0.8777	3.49	29.90	−3.125 10^−1^	4.279	5.717 10^−2^
25/60	50	60	0.4167	0.0495	0.2567	1.4850	3.94				
25/80	50	80	0.3125	0.0697	0.3387	2.0909	4.16				
25/100	50	100	0.2500	0.0890	0.5447	2.6692	4.25				

30/40	60	40	0.7500	0.0279	0.1900	0.8361	3.33	29.34	−3.243 10^−1^	3.918	2.034 10^−2^
30/60	60	60	0.5000	0.0481	0.1559	1.4425	3.83				
30/80	60	80	0.3750	0.0682	0.2167	2.0464	4.07				
30/100	60	100	0.3000	0.0866	0.3731	2.5991	4.14				
30/120	60	120	0.2500	0.1064	0.4731	3.1922	4.23				

35/60	70	60	0.5833	0.0461	0.1642	1.3833	3.67	30.24	−4.157 10^−1^	2.157	1.579 10^−2^
35/80	70	80	0.4375	0.0674	0.1754	2.0210	4.02				
35/100	70	100	0.3500	0.0873	0.2003	2.6200	4.17				
35/120	70	120	0.2917	0.1067	0.2997	3.1997	4.24				

40/60	80	60	0.6667	0.0454	0.1376	1.3633	3.62	29.80	−4.194 10^−1^	2.002	2.061 10^−2^
40/80	80	80	0.5000	0.0653	0.1864	1.9601	3.90				
40/100	80	100	0.4000	0.0859	0.2221	2.5781	4.10				
40/120	80	120	0.3333	0.1052	0.2539	3.1557	4.19				
40/140	80	140	0.2857	0.1248	0.3040	3.7455	4.26				

Ornytion 107A											
20/40	40	40	0.5000	0.4809	0.2739	0.9617	3.83	30.19	−2.406 10^−1^	13.24	−2.675 10^−1^
20/50	40	50	0.4000	0.6396	0.3707	1.2792	4.07				
20/60	40	60	0.6667	0.7827	0.5387	1.5654	4.15				

25/40	50	40	0.6250	0.4477	0.1479	0.8954	3.56	30.02	−2.955 10^−1^	8.317	−1.682 10^−1^
25/60	50	60	0.4167	0.7584	0.3513	1.5168	4.02				
25/80	50	80	0.3125	1.0571	0.5058	2.1141	4.21				
25/100	50	100	0.2500	1.3489	0.6509	2.6978	4.29				

30/40	60	40	0.7500	0.4313	0.0702	0.8625	3.43	29.43	−3.003 10^−1^	6.252	−1.867 10^−1^
30/60	60	60	0.5000	0.7363	0.1824	1.4727	3.91				
30/80	60	80	0.3750	1.0397	0.2957	2.0795	4.14				
30/100	60	100	0.3000	1.3143	0.4646	2.6285	4.18				
30/120	60	120	0.2500	1.6139	0.5543	3.2278	4.28				

35/60	70	60	0.5833	0.7074	0.1848	1.4148	3.75	29.74	−3.497 10^−1^	2.086	6.902 10^−2^
35/80	70	80	0.4375	1.0275	0.2796	2.0549	4.09				
35/100	70	100	0.3500	1.3173	0.2182	2.6347	4.19				
35/120	70	120	0.2917	1.6022	0.3444	3.2044	4.25				

40/60	80	60	0.6667	0.6794	0.1500	1.3588	3.60	30.04	−4.254 10^−1^	1.910	3.058 10^−2^
40/80	80	80	0.5000	0.9936	0.1902	1.9873	3.95				
40/100	80	100	0.4000	1.3001	0.2174	2.6003	4.14				
40/120	80	120	0.3333	1.5906	0.2359	3.1812	4.22				
40/140	80	140	0.2857	1.8830	0.3191	3.7659	4.28				

**Table 3:** Parameters *k*_*D*_ and *δ* from the fittings of the proposed analytical model (with *ε* = *C*_*mf*_ = 0, see expression (24)), to the testing results (see Figure 6). The table also includes the slope, d*K*_*S*_/d*r*_*r*_, the offset, *K*_*S*0_, and determination coefficient *R* ^2^ of the linear fittings for each case.

*R*_*rc*_ [mm]	*k*_*D*_	*δ*	*K*_*S*0_ (=*K*_*A*_)	d*K*_*S*_/d*r*_*r*_ (=*K*_*c*_ )	*R* ^2^
Climatronics 100075					
40	0.6450	−1.7618	4.4353	−1.4874	0.9961
60	0.6505	−1.6958	4.5983	−1.5287	0.9747
80	0.6481	−1.5494	4.5779	−1.3297	0.9758
100	0.6372	−1.1801	4.4256	−0.8035	0.6975
120	0.6343	−0.9889	4.3904	−0.5810	0.6002

Ornytion 107A					
40	0.6544	−1.7731	4.5936	−1.5784	0.9635
60	0.6570	−1.7327	4.7082	−1.6406	0.9972
80	0.6511	−1.5238	4.6200	−1.2895	0.9521
100	0.6418	−1.2409	4.4985	−0.9116	0.8076
120	0.6391	−1.0947	4.4661	−0.7407	0.9999

**Table 4:** Fitting coefficients of the linear approximation to parameters *K*_*A*_ and *K*_*c*_ (see expressions (55)) from the Climatronics 100075 and Ornytion 107A anemometer testing campaign, as a function of *R*_*rc*_ for *R*_*rc*_ > 60 mm. See also Figure 7.

Anemometer	*K*_*A*0_	*K*_*AS*_ [mm^−1^]	*K*_*c*0_	*K*_*cS*_ [mm^−1^]
Climatronics 100075	4.8473	−0.0039	−2.5769	0.0168
Ornytion 107A	4.9548	−0.0042	−2.5305	0.0154

## Appendices

### A.

Anemometer calibrations are performed in the S4 wind tunnel at the IDR/UPM Institute. This is an open-circuit wind tunnel with a closed test section measuring 0.9 by 0.9 m. It is served by four 7.5 kW fans with a flow uniformity under 0.2% in the testing area. The wind speed in the testing chamber is measured by Airflow 0.48 Pitot tube connected to a Druck LPM 9481 high-precision pressure transducer, with the electrical signal from the pressure transducer measured by a Keithley 2000 digital multimeter. Temperature and humidity sensors (Vaisala PTU 200 and Vaisala HMP45D) are used to determine the air density value. The rotation frequency of the anemometer is measured with an Agilent 53131A universal counter. Another digital multimeter is used to measure the voltage or current output from the anemometer when required.

An example of the calibrations performed on this wind tunnel is included in Figure 10. The wind speed measured by the wind tunnel instruments, *V*, is plotted in comparison to the anemometer's frequency output, *f*. The calibration curve (i.e., the linear transfer function) corresponding to the AC calibration is plotted in the aforementioned figure (its mathematical expression is also included). The uncertainty levels of the calibrations performed at the S4 calibration wind tunnel are specified following the ISO/IEC 17025 standard [137], these levels being 0.1 m s^−1^ for wind speeds from 4 m s^−1^ to 10 m s^−1^ and 0.01 *V* m s^−1^ for wind speeds, *V*, from 10 m s^−1^ to 23 m s^−1^.

### B.

The aim of this Appendix is to find the roots of equation:

(B.1) $$\begin{array}{c} \Omega^{2}-2\Omega \gamma \left(1+a\right)+1+b=0 \end{array}$$

in the case *a*, *b* ≪ 1.

If the following perturbation solution is assumed:

(B.2) $$\begin{array}{c} \Omega =\Omega_{0}+\Omega_{1};\;\Omega_{1}\ll \Omega_{0}, \end{array}$$

then

(B.3) $$\begin{array}{c} \Omega_{0}^{2}+2\Omega_{0}\Omega_{1}+\Omega_{1}^{2}-2\gamma \left(\Omega_{0}+\Omega_{1}\right)\left(1+a\right)+1+b=0. \end{array}$$

The solution is as follows, if Ω_1_ = *a* = *b* = 0:

(B.4) $$\begin{array}{c} \Omega_{0}^{2}-2\gamma \Omega_{0}+1=0, \end{array}$$

where

(B.5) $$\begin{array}{c} \Omega_{0}=\gamma -\sqrt{\gamma^{2}-1}=\frac{1-k_{D}}{1+k_{D}}. \end{array}$$

If (B.4) is subtracted from (B.3), taking into account Ω_1_^2^ ≪ 1, the following expression is obtained:

(B.6) $$\begin{array}{c} 2\Omega_{1}\left[\Omega_{0}-\gamma \left(1+a\right)\right]-2\Omega_{0}a\gamma +b=0, \end{array}$$

and then

(B.7) $$\begin{array}{c} \Omega_{1}=\frac{1}{2}\frac{2\Omega_{0}a\gamma -b}{\Omega_{0}-\gamma }=\frac{\Omega_{0}a\gamma -b/2}{\Omega_{0}-\gamma }, \end{array}$$

where

(B.8) $$\begin{array}{c} \Omega_{0}-\gamma =-4\frac{k_{D}}{1-k_{D}^{2}}. \end{array}$$

Once the problem has been linearized, correction Ω_1_ depends on perturbations *a* and *b*.
